# Neurodevelopmental variations cascading from age 10 months to 3 years leading to attention‐deficit hyperactivity disorder/autism traits at age 9 in a general population

**DOI:** 10.1002/jcv2.70053

**Published:** 2025-10-12

**Authors:** Kenji J. Tsuchiya, Nagahide Takahashi, Yoko Nomura, Jeffrey H. Newcorn, Shigenobu Toda, Yuuka Ishikawa‐Omori, Akemi Okumura, Mohammad Shafiur Rahman, Toshiki Iwabuchi, Taeko Harada, Ikue Hirata, Chikako Nakayasu, Yuko Amma, Haruka Suzuki, Tomoko Nishimura

**Affiliations:** ^1^ Research Center for Child Mental Development Hamamatsu University School of Medicine Hamamatsu Japan; ^2^ United Graduate School of Child Development, The University of Osaka, Kanazawa University, Hamamatsu University School of Medicine, Chilba University and University of Fukui Suita Japan; ^3^ Department of Developmental Disorders National Center for Neurology and Psychiatry Tokyo Japan; ^4^ Department of Psychology Queens College and Graduate Center City University of New York New York New York USA; ^5^ Department of Psychiatry Icahn School of Medicine at Mount Sinai New York New York USA; ^6^ Department of Environmental Medicine Icahn School of Medicine at Mount Sinai New York New York USA; ^7^ Department of Psychiatry Shizuoka Psychiatric Medical Center Shizuoka Japan; ^8^ Department of Psychiatry Showa Medical University Tokyo Japan; ^9^ Department of Neuropsychiatry and Behavioral Sciences Kanazawa University School of Medicine Kanazawa Japan; ^10^ Kanagawa University of Human Services Graduate School of Health Innovation Kawasaki Japan

**Keywords:** attention deficit hyperactivity disorder, autism spectrum disorder, Birth Cohort, developmental coordination disorder, intellectual developmental disorder, neurodevelopment

## Abstract

**Background:**

Neurodevelopmental delay precedes attention‐deficit/hyperactivity disorder (ADHD) and autism spectrum disorder (ASD) diagnoses. However, exactly when this emerges in association with these outcomes remains unclear. We investigated (1) the earliest representations of neurodevelopmental variation and (2) what neurodevelopmental variations at an early age (10 months to 3 years) are specifically associated with the emergence of parentally reported behavioral traits of ADHD, ASD, and related disorders at age 9.

**Methods:**

Children from the Hamamatsu Birth Cohort of Mothers and Children (HBC Study) born between December 2007 and March 2012 were enrolled and followed from birth to age 9. Standardized scores of neurodevelopment across five domains (gross motor, visual reception, fine motor, receptive language, and expressive language) were calculated using the Mullen Scales of Early Learning at ages 10, 14, 18, 24, 32, and 40 months. ADHD and ASD traits were ascertained as *Z*‐scores using the ADHD‐Rating Scale and Social Responsiveness Scale‐2 at age 9. To explore the earliest recognition of neurodevelopmental variation with credibility, structural equation modeling (SEM) was used to estimate the total effects of the linear associations between exposures and outcomes.

**Results:**

836 children (405 females, 48%) were enrolled in the analysis. The ADHD trait was associated with visual reception score before age 1 and was concurrent with both fine motor and receptive language scores between ages 1–2. The ASD trait was associated with gross motor score before age 1 and expressive language score between ages 1.5 and 3.

**Conclusion:**

Suboptimal visual‐motor coordination can be a prototype of the ADHD trait, while a cascading pattern of suboptimalities from gross motor to language domains can be a prototype of the ASD trait. The distinct neurodevelopmental variations related to ADHD and ASD traits running in the general population can be found before age 1.

## Key Points

**What's known?**

- Neurodevelopmental delay during infancy and early childhood, including motor delay, is associated with attention‐deficit/hyperactivity disorder (ADHD) and autism spectrum disorder (ASD) traits, although the findings are inconsistent.
- The earliest timing of the emergence of the delay is not precisely known.

**What's new?**

- In a representative birth cohort of a general population (*N* = 836), variations in visual reception before age 1 and in both fine motor and receptive language between ages 1–2 were associated with the ADHD trait at age 9.
- Variations in gross motor before age 1 and in expressive language between ages 1.5 and 3 were associated with the ASD trait at age 9.

**What's relevant?**

- The earliest representations of ADHD and ASD traits can be recognized before age 1, cascading separate sets of suboptimal neurodevelopment.

## Graphical abstract

Associations of neurodevelopmental measures (*Z*‐scores of gross motor, visual reception, fine motor, receptive language, expressive language) at ages 10, 14, 18, 24, 32, and 40 months with (a) attention‐deficit/hyperactivity disorder trait, (b) autism spectrum disorder trait, (c) developmental coordination disorder trait, and (d) intellectual developmental disorder trait at age 9 years.

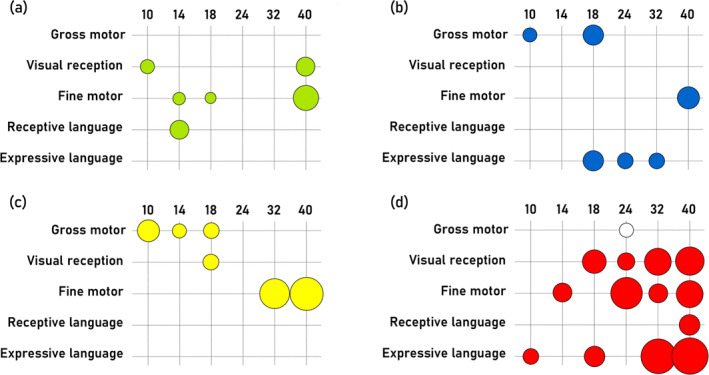

## INTRODUCTION

Attention‐deficit/hyperactivity disorder (ADHD) and autism spectrum disorder (ASD) are defined in the Diagnostic and Statistical Manual of Mental Disorder, 5th Edition Text Revision (DSM‐5‐TR): ADHD is characterized by inattention and hyperactivity, while ASD is characterized by social impairments and repetitive and restricted patterns of behaviors (American Psychiatric Association, 2022). Despite the differences, distinguishing between the two conditions remain challenging, as they often coexist (Shoaib et al., 2022). Both belong to a supra‐category of neurodevelopmental disorders (NDDs) that have common features of atypical neurodevelopment and poor functioning in early childhood (Finlay‐Jones et al., 2019; Thapar & Rutter, 2015). In particular, the neurobiological changes occurring in children with ADHD and/or ASD may involve overlapping features, often referred to as endophenotypes (Johnson et al., 2015). Moreover, suboptimal neurodevelopmental patterns, when considered as candidate early markers of NDDs, can be confirmed well before formal diagnoses (Kaczkurkin et al., 2020).

Recent studies examining the underlying pathophysiology of ADHD and/or ASD have focused on non‐specific neurodevelopmental delay, particularly motor skills. Studies have shown that motor difficulties are frequently found in children and adolescents with ADHD and ASD, and that the types of motor difficulties differ between ADHD and ASD (Ament et al., 2015; Biscaldi et al., 2015; De Francesco et al., 2023). In turn, motor delay in infancy can serve as the earliest representation of NDDs, the ASD trait in particular (Estes et al., 2015; Iverson et al., 2019; Ozonoff et al., 2014), a protype cascading suboptimal neurodevelopment across more than one neurodevelopmental domains (Athanasiadou et al., 2020; Bowler et al., 2024; Lim et al., 2021; Shephard et al., 2022; West, 2019). Motor skills are divided into two domains: the gross motor domain of body movements using major skeletal muscles and the fine motor domain of hand and finger movements that require visual processing (Mullen, 1995). An association of gross motor delay with ADHD was indicated, although the effect size was small (Bowler et al., 2024), whereas the association of gross motor delay with ASD was consistent and the effect size became larger as the child became older (≥ age 2) (Lim et al., 2021; West, 2019). Inconsistencies remain in studies of the association of fine motor skills with ADHD and ASD symptoms or diagnoses (Havmoeller et al., 2019; Kaiser et al., 2015; Lim et al., 2021). This suggests that early motor skills are not directly related to outcomes such as ADHD and/or ASD but instead connected with delays in different neurodevelopmental domains that cascade with age.

To analyze this, we need further research in several areas. First, the specific timing of when neurodevelopmental delay occurs. What are the earliest representations? Can we confirm the association of neurodevelopmental delay to outcomes at specific ages? This requires: (1) A longitudinal design with a large sample of children in early childhood. Trajectory studies based on mixed modeling (Nishimura et al., 2019; Nishimura et al., 2016) provided statistical indices such as slope and intercept. However, these statistics have not elucidated the exact timing of the suboptimality. Additionally, correlations among a series of measures of neurodevelopmental variations measures should be taken into consideration; without such a consideration, the magnitude of the associations of neurodevelopmental delay with the later emergence of ADHD and/or ASD may be overestimated along with age. To address this issue, structural equation modeling (SEM) is needed instead of an iteration of independent regression analyses. (2) Cross‐domain specificity. It has been suggested that delay in motor skill‐related domains—visuomotor and audiomotor integration and language in particular (Bhat, 2023; Carsone et al., 2021; Hadar et al., 2021; Kaiser et al., 2015; Tirosh et al., 2006)—are associated with ADHD, ASD, and other NDDs (Nishimura et al., 2019; Nishimura et al., 2016; Takahashi et al., 2020). To examine this delay, standardized child composite scales to measure gross and fine motor domains, as well as visual and language domains, throughout childhood are needed (Mullen, 1995). (3) An examination of outcome‐specificity. Are neurodevelopmental domains associated with either ADHD or ASD, or both? Considering that the majority of children with a clinical diagnosis of a specific NDD have a co‐existing trait of another NDD diagnosis (Hong et al., 2021; Shoaib et al., 2022), we would expect dimensional measures of ADHD and ASD traits to be the outcome of interest instead of a formal diagnosis. Indeed, studies based on clinical diagnoses of ADHD and/or ASD might have obscured the trait‐specific relevance of neurodevelopment to the emergence of the ADHD and ASD traits (Goulardins et al., 2017; Havmoeller et al., 2019). This approach would further allow us to detect patterns of suboptimal neurodevelopmental variations leading to multiple traits of NDDs, as well as traits of other overlapping NDDs, such as developmental coordination disorder (DCD) and intellectual developmental disorder (IDD) (Verbecque et al., 2025).

This study followed a large general population sample from infancy to late childhood to examine whether early neurodevelopmental variations from age 10 months to 3 years are linearly associated with parentally reported behavioral traits of ADHD and/or ASD at age 9. We also examined whether the variations could also be related to the DCD and IDD traits as reference.

## METHODS

### Participants

A total of 836 infants born between December 24, 2007, and March 9, 2012, who originally participated in the Hamamatsu Birth Cohort for Mothers and Children (HBC Study) (Takagai et al., 2016; Tsuchiya et al., 2010), and were followed up to age 9, were regarded eligible and thus included in the analysis. All the participating children were born in the university hospital of the Hamamatsu University School of Medicine. 99% of the participating children speak Japanese as their first language. Participating children included 80 sibling pairs (14 twin pairs) and 1 set of a sibling trio, which were accounted for in the SEM analysis using robust standard errors.

### Measures of interest

The ADHD, ASD, DCD, and IDD traits were ascertained as dimensional measures of outcome at age 9 using the ADHD‐Rating Scale (ADHD‐RS), Social Responsiveness Scale‐2 (SRS‐2), Developmental Coordination Disorder Questionnaire (DCDQ), and the Wechsler Intelligence Scale for Children, 4th version (WISC‐IV). All the rating scales have been standardized for Japanese children (Kamio et al., 2013; Nakai et al., 2011; Nihonban WISC‐IV Kankō Iinkai, 2010; Tanaka et al., 2016). To compare across measures, we made a single index from each of the scales: standardized percentile score of ADHD‐RS (range 0 to 100) for the measure of the ADHD trait, standardized total *T*‐score of SRS‐2 (range 40 to 90) for the ASD trait, DCDQ total score (range 15 to 75) for the DCD trait, and full‐scale IQ (range 47 to 145) as measured by the WISC‐IV for the IDD trait. We further converted these scores into *Z*‐scores with a mean of 0 and standard deviation of 1. In addition, high scores on the ADHD‐RS and SRS‐2 represented suboptimal outcomes, with the DCDQ and WISC‐IV scales reflecting the opposite. For comparability, we reversed the signs of ADHD and ASD trait scores. This gave us four *Z*‐converted measures of ADHD, ASD, DCD, and IDD traits at age 9, with a lower score indicating more prone to suboptimality, to use in the analyses. The correlation matrix of the trait measures is provided in Table S1. The distribution of the trait measures is provided in Figure S1.

We measured the neurodevelopmental variations of the participating children at approximately 10, 14, 18, 24, 32, and 40 months after birth using the Mullen Scales of Early Learning (MSEL) (Mullen, 1995). The MSEL provides standardized scores in five domains of neurodevelopment (gross motor, visual reception, fine motor, receptive language, and expressive language). The mean standard *T*‐score of each domain for every month between 1 and 50 after birth was set at 50, with an SD of 10, standardized for Japanese children (Nishimura et al., 2016). The MSEL assessment was conducted through a face‐to‐face interview by well‐trained examiners blind to the previous status of the assessments. This gave us *T*‐scores of the five domains for six time points (10, 14, 18, 24, 32, and 40 months). Examples of items included in the MSEL are shown in Table S2. The *T*‐scores were further converted to *Z*‐scores with a mean of 0 and standard deviation of 1 throughout the analyses below. The distribution of the *Z*‐converted domain scores is provided in Figure S2.

Child's sex, birth order, and maternal educational achievement in years were included in the analyses as covariates as they showed potential associations with neurodevelopment, related to the ADHD, ASD, DCD, and IDD traits.

### Analytical plan

**FIGURE 1:**
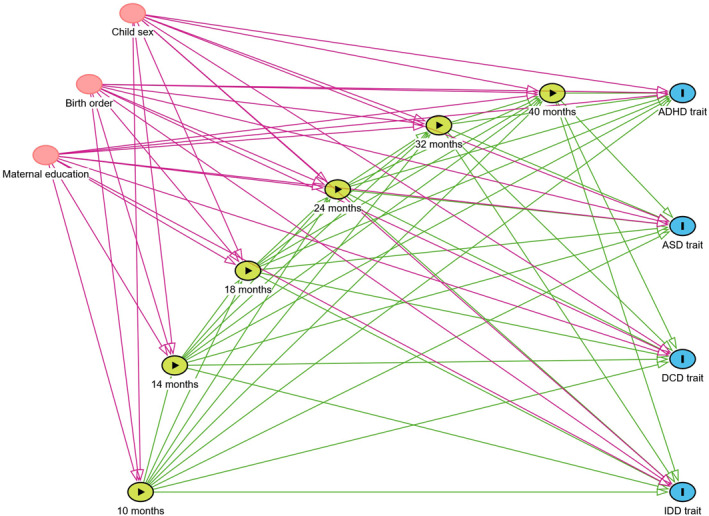
The analyses plan of this study using a directed acyclic graph. Each observation through 10–40 months (green ovals with triangles, exposure variables) involves face‐to‐face observation of developmental measures in domains of gross motor, visual reception, fine motor, receptive language, and expressive language using Mullen Scales of Early Learning. The *T*‐scores provided of the five domains were converted to *Z*‐score before analyses. For the ease of comprehension, we denoted each observation as one oval, although the actual analyses were conducted in the manner that all the five *Z*‐scores from the five neurodevelopmental domains were separately entered into the model. The outcome variables (blue ovals with I) involve attention‐deficit/hyperactivity disorder, autism spectrum disorder, DCD, and IDD trait scores. Potential confounding was considered: three covariates (child sex, birth order, maternal educational history in years, shown in pink ovals) were adjusted for in every association between any of the exposure and of outcome variable that emerged in this structural equation modeling. DCD, developmental coordination disorder; IDD, intellectual developmental disorder.

To minimize the number of statistical tests as well as handle missingness (approximately 5% to 10% occurring at each wave), SEM with the full‐information maximum likelihood option, with robust standard errors applied for a consideration of clustering effects of siblings and twins, was applied to the analyses. Linear associations between the neurodevelopmental measures and the outcomes of interest were assessed using SEM in four separate longitudinal models. Neurodevelopmental measures in five domains at any age were assumed to predict all the *Z*‐scores of all the domains as the child grew. As such, all the possible paths across the domains and timings are drawn. A summary scheme of the analysis is shown in a directed acyclic graph (Figure 1). As we were not interested in segregating direct and indirect pathways leading to a trait of specific NDD, we reported total effects based on standardized regression coefficients with robust standard errors, adjusted for covariates. This also allowed us to account for any clustering effect of siblings or twins. The cutoff for two‐sided *p*‐values was set at 0.05. All analyses were conducted using Stata version 18.0 (Stata Corp).

## RESULTS

### Participants and sample

Table 1 shows the characteristics of the participating children (*N* = 836). We observed no predominance in female or male participants, history of premature birth, or underachievement in maternal educational history. The overall sample characteristics corresponded well with Japanese children of equivalent ages: the ADHD, ASD, DCD, and IDD trait scores showed no departure from the population norm (Table 2).

**TABLE 1:** Characteristics of the participating children (*N* = 836).

	*N* (%) or mean (SD)
Demographic data	
Number of children	836
Child sex (female)	405 (48%)
Birth order (first‐born)	420 (50%)
Gestational age at birth (weeks)	38.9 (1.6)
Prematurity (<37 weeks)	62 (7%)
Maternal education (years)	14.0 (1.9)
Behavioral representations at age 9	
ADHD trait (ADHD‐RS, total percentile score before conversion to inverted *Z*‐score)	51.8 (29.0)
ASD trait (SRS‐2, standardized total *T*‐score before conversion to inverted *Z*‐score)	51.0 (9.9)
DCD trait (DCDQ, total score before conversion to *Z*‐score)	57.7 (10.0)
IDD trait (WISC‐IV, full‐scale IQ before conversion to *Z*‐score)	101.0 (14.0)

Abbreviations: ADHD, attention‐deficit/hyperactivity disorder; ASD, autism spectrum disorder; DCD, developmental coordination disorder; IDD, intellectual developmental disorder; WISC‐IV, the Wechsler Intelligence Scale for Children, 4th version.

**TABLE 2:** Neurodevelopmental domain scores of the participating children at 10, 14, 18, 24, 32, and 40 months (*N* = 836).

	Mean (SD)					
Neurodevelopmental domains	10 months	14 months	18 months	24 months	32 months	40 months
Gross motor at 10 months (*N* = 797)	46.9 (9.6)	48.3 (10.6)	49.2 (9.8)	48.7 (10.0)	49.0 (10.3)	49.7 (10.0)
Visual reception at 10 months (*N* = 796)	48.3 (10.6)	48.8 (9.6)	48.7 (9.7)	49.6 (10.3)	49.1 (9.5)	50.2 (10.3)
Fine motor at 10 months (*N* = 796)	48.1 (10.2)	48.8 (9.8)	49.5 (9.8)	48.4 (9.7)	49.4 (10.2)	50.8 (10.3)
Receptive language at 10 months (*N* = 794)	48.0 (10.0)	49.7 (9.7)	48.7 (9.7)	50.1 (10.0)	49.7 (9.3)	50.0 (9.8)
Expressive language at 10 months (*N* = 788)	47.9 (9.6)	49.3 (10.3)	48.8 (9.3)	49.4 (9.8)	49.2 (9.9)	50.0 (10.2)

*Note*: *T*‐scores of Mullen Scales of Early Learning with a population mean set of 50 and SD of 10 before conversion to *Z*‐score.

### Main analysis

**FIGURE 2:**
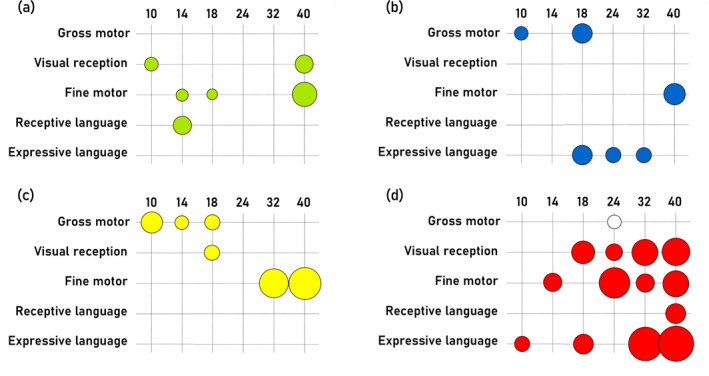
Associations of neurodevelopmental measures (*Z*‐scores of gross motor, visual reception, fine motor, receptive language, expressive language) at ages 10, 14, 18, 24, 32, and 40 months with (A) ADHD trait, (B) ASD trait, (C) DCD trait, and (D) IDD trait at age 9 years. Circles with colors indicate statistically significant associations with *p* < .05. Size of the circles represent standardized regression coefficients. Hollow circles indicate inverse associations. (A) ADHD trait (green): inverted *Z*‐converted ADHD‐RS percentile score. (B) ASD trait (blue): inverted *Z*‐converted SRS‐2 total *T*‐score. (C) DCD trait (yellow): *Z*‐converted Developmental Coordination Disorder Questionnaire total score. (D) IDD trait (red): *Z*‐converted full‐scale IQ. ADHD, attention‐deficit/hyperactivity disorder; ASD, autism spectrum disorder; DCD, developmental coordination disorder; IDD, intellectual developmental disorder.

Figure 2A shows that a gross motor domain score was not associated with the ADHD trait at age 9. However, scores in visual reception (10 and 40 months), in fine motor (14, 18, 40 months), and in receptive language (14 months) domains were associated with the ADHD trait at age 9.

Figure 2B shows that scores in gross motor (10 and 18 months), in fine motor (40 months), and in expressive language (18, 24, 32 months) domains were associated with the ASD trait at age 9.

Figure 2C shows that scores in gross motor (10, 14, 18 months), in visual reception (18 months), and in fine motor (32, 40 months) domains were associated with the DCD trait at age 9.

Figure 2D shows that scores in visual reception (18, 24, 32, 40 months), in fine motor (14, 24, 32, 40 months), in receptive language (40 months) and in expressive language (10, 18, 32, 40 months) domains were associated with the IDD trait (*Z*‐score of full‐scale IQ) at age 9. Gross motor score at 24 months were inversely associated with the IDD trait.

The full results of the findings above are presented in Table S3. Also, the full results of the analysis using separate linear regression analyses instead of SEM are presented in Table S4. To see whether children with a suboptimal neurodevelopmental status potentially exhibited clinical concerns, proportions of children with subthreshold ADHD, ASD, DCD, and IDD trait scores (threshold set at −1.5 SD at age 9) by neurodevelopmental status (< −1SD, within ± 1SD, > +1SD) in five neurodevelopmental domains at ages 10–40 months are presented in Table S5.

## DISCUSSION

### Main findings

Based on our longitudinal study, with its large representative sample, we found that (1) variations in early (<age 2) visual reception, in fine motor and receptive language between ages 1–2, and in late (≥age 3) visual reception, were specifically associated with the ADHD trait at age 9; (2) variations in early (<age 2) gross motor and expressive language between ages 1.5–3 were specifically associated with the ASD trait at age 9; (3) variation in late (≥age 3) fine motor was associated with ADHD, ASD, DCD, and IDD traits. As expected, gross motor and fine motor were both associated with the DCD trait, and neurodevelopmental variation in all the domains except for gross motor was associated with the IDD trait (IQ). Considering the direction of the linear associations, early visual reception suboptimality followed by fine motor and receptive language suboptimalities were associated with an elevated level of the ADHD trait, and early gross motor suboptimality followed by expressive language suboptimality were associated with an elevated level of the ASD trait.

### Suboptimal neurodevelopmental variations leading to the ADHD trait

Our study did not support an association of early gross motor delay with the ADHD trait, unlike an early study (Gurevitz et al., 2014). Explanations for the inconsistency include lack of consideration of DCD in children diagnosed for ADHD in early studies (Havmoeller et al., 2019), and a reflection of a concurrent link between hyperactivity and activities involving gross motor skills (Goulardins et al., 2017). In the present study, an inverse association of gross motor development, though insignificant, was observed at 14 months (Table S1)—an inverse direction of gross motor influence before age 1 (Havmoeller et al., 2019)—implying that a link between early gross motor skills and the ADHD trait is not supported in a general population.

In turn, fine motor delay, measured with activities that involve visual information processing coordinated with motor skills (Goto et al., 2024), has been consistently reported in cross‐sectional and case‐control studies (Kaiser et al., 2015). Our findings were consistent with these, as early fine motor delay before age 2 was associated with the ADHD trait. Beyond the limitation of cross‐sectional and case‐control studies, one prospective study suggested that children who are later diagnosed as ADHD are likely to show fine motor delay as early as age 1 (Gurevitz et al., 2014). The present study is in line with the above finding.

Our analysis found that visual reception delay, which involves the ability to decode visual input, during very early life (<age 1) and later (>age 3) was associated with the ADHD trait. Furthermore, we found a progressive pattern of very early visual reception delay leading to fine motor delay between ages 1–2. We assume these progressive patterns will also affect development of visual‐motor integration (VMI). VMI skills emerge in early childhood and are a key component leading to meaningful activities (Dankert et al., 2003). In composite scales like MSEL, the fine motor domain can capture VMI skills in items such as “imitates four‐block train using blocks” (Mullen, 1995). Of note, delays in both visual reception and fine motor domains after age 1 are consistently associated with the IDD trait (lower IQ) in the present study; this is in line with studies including IDD that have reported difficulties in VMI (Carsone et al., 2021), and point to the relevance of early suboptimal VMI skills as a prototype of the ADHD and IDD traits later in childhood.

One meta‐analytic study suggested that language delay below age 5 is associated with an ADHD diagnosis (Shephard et al., 2022). However, the studies included in the meta‐analysis did not separate the influence derived from co‐existing conditions such as ASD, raising the possibility that the association between language delay and an ADHD diagnosis might reflect a co‐existing ASD trait: our data supports this interpretation.

We can also present a novel finding: neurodevelopmental delay specifically in receptive language development at only 14 months is uniquely associated with the later ADHD trait. This may reflect underlying attention network development (Posner et al., 2016), as receptive language skills require sustained attention to others' verbal output and aligns with theoretical frameworks suggesting that early executive dysfunction, particularly in attention and response regulation, represents a core feature of ADHD (Christoforou et al., 2023; Posner et al., 2016).

### Suboptimal neurodevelopmental variations leading to the ASD trait

In line with early reviews (Lim et al., 2021; West, 2019), early gross motor delay—“a base for learning” in early childhood (Mullen, 1995)—was associated with the ASD trait. However, the association was supported only before age 2. The most likely explanation for no association for ≥ age 2 is that we ran SEM to consider earlier neurodevelopmental measures that might explain later ones (Tables S3 and S4). This left the possibility that gross motor domain scores at ≥ age 2 are associated with the ASD trait if the scores before age 2 are not considered and that our study is not necessarily inconsistent with early studies. Nevertheless, our study design allows us to assert that gross motor delay emerging as early as < age 1 can be the earliest presentation of the ASD trait seen later in life.

In MSEL, language skills are measured in two domains: receptive and expressive language. The receptive language domain concerns the ability to respond to and decode other's verbal outputs, whereas the expressive language domain covers the auditory processes coupled with the use of vocal responses and spoken language (Mullen, 1995). Early studies have suggested that language delay is associated with the emergence of ASD symptoms or diagnosis later in life (Hansen et al., 2008; Zwaigenbaum et al., 2005). Our data provided support for the relevance of delayed expressive language development observed between ages 1.5 to 3, specifically to the ASD trait in a general population. Intriguingly, expressive language delay does not emerge before age 1 and appears to follow the gross motor delay that emerges at 10 months (Figure 2B), which is in line with results of a meta‐analytic study supporting neurodevelopmental delay in both gross motor and expressive language domains in children at an increased risk of ASD (Garrido et al., 2017), and with a longitudinal study showing that gross motor delay precedes expressive language delay (Bedford et al., 2016). We suggest that a progression from an early gross motor delay to expressive language delay is a specific underpinning, or prototype, of the ASD trait.

Visual reception delay was not specifically associated with the ASD trait in our study, despite the existing literature consistently demonstrating the relevance of delayed VMI development in children with ASD (Bhat, 2023; Carsone et al., 2021; Wu et al., 2021). Of note, an early study pointed out that young children with ASD symptoms showed lower scores in VMI skills, although the scores were independent of language skills (Barbeau et al., 2015). Our understanding of this apparent inconsistency is that children with the ASD trait later in life may have concurrent but distinct neurodevelopmental processes of visual‐motor delay and audio‐motor delay, including delayed language production.

### Non‐specific suboptimal neurodevelopmental variations

Fine motor delay at 40 months was related to NDD traits in general. At 40 months, 50% of children are expected to achieve “cuts with scissors” or “draws line in paths” (Mullen, 1995). Clearly, skills that develop at this late stage require visual and auditory processes as well as motor coordination to sensory processes. Since these items do not specifically assess fine motor skills nor VMI skills, we should not treat fine motor skills at 40 months in line with early fine motor skills before age 2.

Of note, the measures for ADHD and ASD traits are significantly correlated with each other with *r* = 0.49 (Table S1), indicating that the variance of one measure accounts for approximately 25% of the other. The overlapping construct of the two traits may be predicted by non‐specific suboptimal neurodevelopmental variations such as fine motor delay at 40 months.

### Clinical implications

The present study provides valuable information for developmental medicine. First, to date the early neurodevelopmental indicators leading to ADHD are not well understood (Visser et al., 2016). Our results imply that it is possible to identify children as young as age 2 who are later found to have possible ADHD. This highlights the importance of transdiagnostic observation and flexible intervention strategies that accommodate behavioral profiles, rather than relying solely on categorical diagnoses in early childhood. Second, studies have argued whether gross motor delay found in children with ADHD may or may not be accounted for by co‐existing DCD (Goulardins et al., 2015; Goulardins et al., 2017). Since our data suggest that gross motor delay was not associated with the ADHD trait, early gross motor delay found in children who later have an ADHD diagnosis are likely to develop DCD trait. Third, since our study design is free from clinical diagnostic categories, an early neurodevelopmental course leading to multiple NDD presentations can be extracted simply by superimposing the neurodevelopmental courses for each of the specific NDD traits. For instance, gross motor delay together with visual reception delay before age 1 and without delay in any other domain may suggest a later emergence of both ADHD and ASD traits; the additional observation of delays in gross motor, fine motor, and language domains between ages 1–1.5, further supports the likelihood of the emergence and co‐occurrence of the two traits.

### Limitations

Since our findings are based on the total effects of SEM models, with earlier neurodevelopmental measures explaining later ones, and since our outcomes of interest were not clinical diagnoses, the assessment of predictive validity for clinical populations using the developmental measures was not allowed. Indeed, the associations we found were linear in nature and the proportion of children who showed ASD trait score below −1.5SD among those with neurodevelopmental scores below −1SD was approximately 10%–20% (Table S5). Also, the assessments of the NDD traits were parent‐reported and questionnaire‐based, which might have introduced information biases.

## CONCLUSION

Early suboptimal neurodevelopmental variations or delay, as early as age 1, are connected with ADHD and ASD traits in a general population. Trait‐specific cascading patterns leading to the ADHD trait can be seen in visual motor integration skills: early visual reception delay, before age 1, is followed by delays in fine motor and receptive language domains before age 2. The corresponding pattern leading to the ASD trait was shown in a progressive pattern in motor and language development: gross motor delay before age 1 followed by expressive language delay between ages 1.5 to 3.

## AUTHOR CONTRIBUTIONS

**Kenji J. Tsuchiya**: Conceptualization; methodology—protocol; data curation; formal analysis; investigation; writing—original draft, reviewing and editing; funding acquisition. **Nagahide Takahashi**: Conceptualization; methodology—reviewing; investigation; writing—original draft, reviewing and editing. **Yoko Nomura, Jeffrey H. Newcorn, Shigenobu Toda**: Methodology—reviewing; investigation—interpretation of the data; writing—reviewing and editing. **Yuuka Ishikawa‐Omori, Akemi Okumura, Mohammad Shafiur Rahman**: Methodology—reviewing; data curation; writing—reviewing and editing; funding acquisition. **Toshiki Iwabuchi, Taeko Harada, Ikue Hirata, Chikako Nakayasu, Yuko Amma, Haruka Suzuki**: Methodology—reviewing; data curation; writing—reviewing and editing. **Tomoko Nishimura**: Conceptualization; methodology—reviewing; data curation; investigation; writing—reviewing and editing; project administration and supervision; funding acquisition. **Tomoko Nishimura** also served as the statistical expert for this research.

## CONFLICT OF INTEREST STATEMENT

The authors declare no conflicts of interest.

## ETHICAL CONSIDERATIONS

The study was conducted in accordance with the guidelines proposed in the World Medical Association Declaration of Helsinki. All the enrolled pregnant individuals were given a complete description of the study and provided written informed consent to participate for both them and their children. The study protocol was approved by the Ethical Committee of Hamamatsu University School of Medicine, Japan (approval number 18–166 on March 14, 2007 and 20–233 on November 5, 2020). The present study conforms to the STrengthening the Reporting of OBservational studies in Epidemiology statement.

## Supporting information

Supporting Information S1

## Data Availability

The consent given by the participants does not allow publicly delivering/sharing data on an individual level in repositories or journals. Researchers who would like to access to HBC Study datasets for replication should apply to tsuchiya@hama-med.ac.jp. Access to the datasets requires approval from HBC Study Executive Committee, Hamamatsu University School of Medicine.
