# Supplementary material for: Neurodevelopmental variations cascading from age 10 months to 3 years leading to attention‐deficit hyperactivity disorder/autism traits at age 9 in a general population
Source: JCPP Adv. 2025 Oct 12;6(2):e70053. doi: 10.1002/jcv2.70053 (PMC13260687; doi:10.1002/jcv2.70053)
Supplement: Supplementary file 1 — Supporting Information S1 [file JCV2-6-e70053-s001.docx]

**Supporting Information**

Table S1. Correlation matrix of the ADHD, ASD, DCD, and IDD traits: correlation coefficients and p-values.

|  | ADHD trait | ASD trait | DCD trait |
| --- | --- | --- | --- |
| ASD trait | .498 (<.001) |  |  |
| DCD trait | .322 (<.001) | .489 (<.001) |  |
| IDD trait | .189 (<.001) | .219 (<.001) | .201 (<.001) |

ADHD trait: inverted Z-converted ADHD-RS percentile score. ASD trait: inverted Z-converted SRS-2 total T-score. DCD trait: Z-converted DCDQ total score. IDD trait: inverted Z-converted full-scale IQ, all measured at age 9 years.

Table S2. Examples of items in the neurodevelopmental domains in the Mullen Scales of Early Learning.^12^

| Items in the gross motor domain that approximately 50% of children are expected to achieve: | |
| --- | --- |
| At age 1  At age 2  At age 3 | “Stands alone”, “Walks alone”  “Walks up stairs by self, non-alternating”  “Balances on one foot” |
| Items in the visual reception domain that approximately 50% of children are expected to achieve: | |
| At age 1  At age 2  At age 3 | “Shows interest in book”, “Attends to picture (in the book)”  “Matches objects without naming”  “Recalls memory of hidden objects” |
| Items in the fine motor domain that approximately 50% of children are expected to achieve: | |
| At age 1  At age 2  At age 3 | “Uses two hands together”  “Stacks more than 6 blocks vertically”  “Copies circle and line” |
| Items in the receptive language domain that approximately 50% of children are expected to achieve: | |
| At age 1  At age 2  At age 3 | “Understands simple commands”  “Understands ‘Put the teddy bear under the table’”  “Follows the command ‘Listen. Give me the ball, then close the book’” |
| Items in the expressive language domain that approximately 50% of children are expected to achieve: | |
| At age 1  At age 2  At age 3 | “Uses a few words”  “Uses two-word phrase”  “Repeats two numbers”, “Uses three- to four-word sentences” |

Table S3. Associations of neurodevelopmental measures (Z-scores of gross motor, visual reception, fine motor, receptive language, expressive language) at ages 10, 14, 18, 24, 32, and 40 months with ADHD, ASD, DCD, and IDD trait scores at age 9 years using structural equation modelling (SEM).

|  | ADHD trait | ASD trait | DCD trait | IDD trait |
| --- | --- | --- | --- | --- |
| Gross motor at 10 months (N=797) | -0.02 [-0.11, 0.06] | **0.09 [0.01, 0.17]** | **0.14 [0.05, 0.22]** | 0.05 [-0.02, 0.13] |
| Visual reception at 10 months (N=796) | **0.09 [0.01, 0.17]** | 0.03 [-0.04, 0.11] | 0.04 [-0.04, 0.12] | 0.08 [-0.00, 0.16] |
| Fine motor at 10 months (N=796) | 0.02 [-0.05, 0.10] | 0.01 [-0.07, 0.09] | 0.07 [-0.01, 0.16] | 0.07 [-0.01, 0.14] |
| Receptive language at 10 months (N=794) | -0.00 [-0.09, 0.08] | -0.01 [-0.10, 0.06] | -0.02 [-0.11, 0.07] | 0.02 [-0.07, 0.11] |
| Expressive language at 10 months (N=788) | 0.01 [-0.07, 0.10] | 0.04 [-0.03, 0.13] | -0.03 [-0.12, 0.06] | **0.10 [0.02, 0.19**] |
| Gross motor at 14 months (N=755) | -0.06 [-0.15, 0.01] | -0.04 [-0.12, 0.04] | **0.09 [0.00, 0.18]** | 0.04 [-0.05, 0.14] |
| Visual reception at 14 months (N=761) | -0.06 [-0.14, 0.01] | -0.01 [-0.09, 0.06] | 0.02 [-0.06, 0.10] | 0.01 [-0.06, 0.10] |
| Fine motor at 14 months (N=760) | **0.08 [0.00, 0.16]** | 0.04 [-0.03, 0.13] | 0.03 [-0.04, 0.10] | **0.12 [0.04, 0.20]** |
| Receptive language at 14 months (N=734) | **0.12 [0.03, 0.21]** | 0.04 [-0.05, 0.15] | 0.01 [-0.08, 0.11] | 0.03 [-0.05, 0.12] |
| Expressive language at 14 months (N=753) | -0.02 [-0.11, 0.07] | 0.08 [-0.01, 0.18] | 0.02 [-0.06, 0.11] | 0.02 [-0.07, 0.11] |
| Gross motor at 18 months (N=801) | 0.06 [-0.02, 0.15] | **0.13 [0.04, 0.23]** | **0.10 [0.02, 0.19]** | 0.04 [-0.03, 0.12] |
| Visual reception at 18 months (N=807) | 0.06 [-0.03, 0.15] | 0.04 [-0.03, 0.13] | **0.10 [0.02, 0.19]** | **0.15 [0.07, 0.24]** |
| Fine motor at 18 months (N=805) | **0.07 [0.00, 0.16]** | 0.00 [-0.08, 0.08] | 0.03 [-0.05, 0.11] | 0.02 [-0.05, 0.10] |
| Receptive language at 18 months (N=792) | 0.04 [-0.05, 0.14] | -0.01 [-0.10, 0.08] | -0.04 [-0.14, 0.06] | 0.03 [-0.06, 0.13] |
| Expressive language at 18 months (N=799) | 0.03 [-0.06, 0.13] | **0.13 [0.04, 0.23]** | 0.08 [-0.02, 0.18] | **0.13 [0.02, 0.24]** |
| Gross motor at 24 months (N=791) | -0.02 [-0.10, 0.06] | 0.00 [-0.08, 0.08] | 0.04 [-0.04, 0.13] | **-0.09 [-0.17, -0.02]** |
| Visual reception at 24 months (N=801) | 0.07 [-0.02, 0.16] | 0.01 [-0.06, 0.09] | 0.03 [-0.05, 0.12] | **0.11 [0.02, 0.20]** |
| Fine motor at 24 months (N=802) | 0.01 [-0.08, 0.09] | 0.08 [-0.01, 0.17] | 0.06 [-0.03, 0.15] | **0.20 [0.13, 0.28**] |
| Receptive language at 24 months (N=778) | 0.07 [-0.03, 0.17] | 0.01 [-0.06, 0.10] | 0.02 [-0.07, 0.11] | 0.05 [-0.04, 0.14] |
| Expressive language at 24 months (N=785) | 0.04 [-0.09, 0.13] | **0.10 [0.00, 0.22]** | 0.04 [-0.07, 0.15] | 0.08 [-0.02, 0.18] |
| Gross motor at 32 months (N=776) | -0.09 [-0.19, 0.00] | -0.03 [-0.12, 0.05] | 0.00 [-0.09, 0.10] | -0.01 [-0.09, 0.07] |
| Visual reception at 32 months (N=792) | 0.10 [-0.00, 0.20] | 0.03 [-0.07, 0.13] | 0.01 [-0.10, 0.11] | **0.17 [0.08, 0.25]** |
| Fine motor at 32 months (N=793) | 0.05 [-0.03, 0.15] | 0.08 [-0.01, 0.17] | **0.19 [0.09, 0.29]** | **0.12 [0.04, 0.19]** |
| Receptive language at 32 months (N=789) | 0.02 [-0.09, 0.13] | -0.05 [-0.18, 0.07] | -0.04 [-0.15, 0.06] | 0.09 [-0.00, 0.18] |
| Expressive language at 32 months (N=789) | 0.04 [-0.06, 0.15] | **0.10 [0.00, 0.21]** | -0.03 [-0.14, 0.07] | **0.22 [0.12, 0.31]** |
| Gross motor at 40 months (N=789) | 0.08 [-0.01, 0.18] | 0.09 [-0.01, 0.20] | 0.08 [-0.01, 0.18] | -0.07 [-0.16, 0.03] |
| Visual reception at 40 months (N=803) | **0.12 [0.01, 0.22**] | 0.01 [-0.10, 0.11] | 0.00 [-0.10, 0.10] | **0.18 [0.09, 0.27]** |
| Fine motor at 40 months (N=805) | **0.16 [0.06, 0.26]** | **0.14 [0.04, 0.24]** | **0.21 [0.11, 0.31]** | **0.17 [0.09, 0.26]** |
| Receptive language at 40 months (N=805) | -0.05 [-0.18, 0.08] | -0.07 [-0.20, 0.06] | 0.01 [-0.12, 0.13] | **0.13 [0.03, 0.24]** |
| Expressive language at 40 months (N=798) | -0.05 [-0.16, 0.06] | -0.03 [-0.14, 0.09] | -0.01 [-0.13, 0.11] | **0.23 [0.13, 0.33]** |

ADHD trait: inverted Z-converted ADHD-RS percentile score. ASD trait: inverted Z-converted SRS-2 total T-score. DCD trait: Z-converted DCDQ total score. IDD trait: inverted Z-converted full-scale IQ, all measured at age 9 years. Bold types indicate the p-values ≤ .05. Child’s sex, birth order, and maternal educational achievement in years were included in the analyses as covariates. The graphical presentations of this table are shown as Figures 2a, b, c, and d.

Table S4. Associations of neurodevelopmental measures (Z-scores of gross motor, visual reception, fine motor, receptive language, expressive language) at ages 10, 14, 18, 24, 32, and 40 months with ADHD, ASD, DCD, and IDD trait scores at age 9 years using linear regression analysis.

|  | ADHD trait | ASD trait | DCD trait | IDD trait |
| --- | --- | --- | --- | --- |
| Gross motor at 10 months (N=797) | 0.01 (-0.07, 0.08) | **0.12 (0.05, 0.20)** | **0.16 (0.09, 0.23)** | **0.09 (0.02, 0.17)** |
| Visual reception at 10 months (N=796) | **0.09 (0.02, 0.15)** | **0.07 (0.00, 0.14)** | **0.10 (0.03, 0.16)** | **0.11 (0.04, 0.17)** |
| Fine motor at 10 months (N=796) | 0.04 (-0.02, 0.11) | 0.06 (-0.01, 0.13) | **0.10 (0.03, 0.17)** | **0.11 (0.04, 0.17)** |
| Receptive language at 10 months (N=794) | 0.03 (-0.04, 0.10) | 0.05 (-0.02, 0.12) | 0.05 (-0.02, 0.12) | 0.04 (-0.03, 0.11) |
| Expressive language at 10 months (N=788) | 0.02 (-0.05, 0.10) | **0.08 (0.00, 0.15)** | 0.02 (-0.05, 0.09) | **0.11 (0.04, 0.18)** |
| Gross motor at 14 months (N=755) | -0.02 (-0.09, 0.05) | 0.05 (-0.02, 0.12) | **0.15 (0.08, 0.21)** | **0.12 (0.06, 0.19)** |
| Visual reception at 14 months (N=761) | -0.01 (-0.08, 0.06) | 0.06 (-0.02, 0.13) | **0.09 (0.02, 0.16)** | **0.10 (0.02, 0.17)** |
| Fine motor at 14 months (N=760) | **0.09 (0.01, 0.16)** | **0.10 (0.02, 0.17)** | **0.09 (0.02, 0.16)** | **0.17 (0.10, 0.24)** |
| Receptive language at 14 months (N=734) | **0.11 (0.03, 0.18)** | **0.09 (0.02, 0.17)** | 0.06 (-0.01, 0.14) | **0.08 (0.00, 0.15)** |
| Expressive language at 14 months (N=753) | 0.03 (-0.04, 0.10) | **0.13 (0.06, 0.20)** | **0.09 (0.02, 0.16)** | **0.10 (0.03, 0.17)** |
| Gross motor at 18 months (N=801) | **0.09 (0.02, 0.16)** | **0.18 (0.11, 0.25)** | **0.18 (0.11, 0.24)** | **0.15 (0.09, 0.22)** |
| Visual reception at 18 months (N=807) | **0.13 (0.06, 0.20)** | **0.13 (0.06, 0.20)** | **0.17 (0.10, 0.24)** | **0.24 (0.17, 0.31)** |
| Fine motor at 18 months (N=805) | **0.13 (0.06, 0.20)** | **0.08 (0.01, 0.15)** | **0.11 (0.04, 0.18)** | **0.15 (0.08, 0.21)** |
| Receptive language at 18 months (N=792) | **0.13 (0.06, 0.21)** | **0.15 (0.08, 0.22)** | **0.12 (0.04, 0.19)** | **0.20 (0.13, 0.27)** |
| Expressive language at 18 months (N=799) | **0.10 (0.02, 0.18)** | **0.22 (0.14, 0.29)** | **0.16 (0.08, 0.23)** | **0.22 (0.15, 0.29)** |
| Gross motor at 24 months (N=791) | 0.05 (-0.02, 0.12) | **0.14 (0.07, 0.21)** | **0.16 (0.09, 0.23)** | **0.11 (0.04, 0.17)** |
| Visual reception at 24 months (N=801) | **0.16 (0.10, 0.23)** | **0.15 (0.08, 0.22)** | **0.16 (0.09, 0.22)** | **0.28 (0.22, 0.34)** |
| Fine motor at 24 months (N=802) | **0.12 (0.05, 0.19)** | **0.17 (0.09, 0.24)** | **0.18 (0.11, 0.25)** | **0.32 (0.25, 0.39)** |
| Receptive language at 24 months (N=778) | **0.18 (0.11, 0.25)** | **0.19 (0.12, 0.26)** | **0.16 (0.08, 0.23)** | **0.25 (0.19, 0.32)** |
| Expressive language at 24 months (N=785) | **0.14 (0.07, 0.21)** | **0.24 (0.17, 0.31)** | **0.19 (0.11, 0.26)** | **0.27 (0.20, 0.34)** |
| Gross motor at 32 months (N=776) | 0.02 (-0.05, 0.09) | **0.12 (0.06, 0.19)** | **0.14 (0.08, 0.21)** | **0.20 (0.13, 0.27)** |
| Visual reception at 32 months (N=792) | **0.20 (0.13, 0.28)** | **0.18 (0.10, 0.25)** | **0.17 (0.10, 0.24)** | **0.41 (0.34, 0.48)** |
| Fine motor at 32 months (N=793) | **0.15 (0.08, 0.22)** | **0.18 (0.11, 0.25)** | **0.25 (0.18, 0.32)** | **0.36 (0.30, 0.42)** |
| Receptive language at 32 months (N=789) | **0.17 (0.10, 0.25)** | **0.15 (0.08, 0.23)** | **0.14 (0.06, 0.21)** | **0.36 (0.28, 0.43)** |
| Expressive language at 32 months (N=789) | **0.14 (0.07, 0.21)** | **0.22 (0.15, 0.29)** | **0.12 (0.05, 0.19)** | **0.38 (0.32, 0.45)** |
| Gross motor at 40 months (N=789) | **0.11 (0.04, 0.19)** | **0.19 (0.12, 0.26)** | **0.20 (0.13, 0.27)** | **0.20 (0.13, 0.27)** |
| Visual reception at 40 months (N=803) | **0.21 (0.14, 0.28)** | **0.14 (0.07, 0.21)** | **0.15 (0.08, 0.21)** | **0.45 (0.39, 0.51)** |
| Fine motor at 40 months (N=805) | **0.22 (0.15, 0.29)** | **0.22 (0.15, 0.29)** | **0.29 (0.23, 0.36)** | **0.45 (0.38, 0.51)** |
| Receptive language at 40 months (N=805) | **0.16 (0.09, 0.24)** | **0.16 (0.08, 0.23)** | **0.17 (0.10, 0.24)** | **0.48 (0.41, 0.54)** |
| Expressive language at 40 months (N=798) | **0.15 (0.08, 0.22)** | **0.18 (0.11, 0.25)** | **0.16 (0.09, 0.23)** | **0.47 (0.41, 0.53)** |

ADHD trait: inverted Z-converted ADHD-RS percentile score. ASD trait: inverted Z-converted SRS-2 total T-score. DCD trait: Z-converted DCDQ total score. IDD trait: inverted Z-converted full-scale IQ, all measured at age 9 years. Bold types indicate the p-values ≤ .05. Child’s sex, birth order, and maternal educational achievement in years were included in the analyses as covariates.

Table S5. Proportion of children with subthreshold ADHD, ASD, DCD, and IDD trait scores (threshold set at -1.5 SD at age 9) by neurodevelopmental status (< -1SD, within +/- 1SD, > +1SD) in five neurodevelopmental domains at 10, 14, 18, 24, 32, and 40 months.

|  |  | Number of children (%) | | | |
| --- | --- | --- | --- | --- | --- |
|  | n | ADHD trait score  below -1.5SD (n=59) | ASD trait score  below -1.5SD (n=62) | DCD trait score  below -1.5SD (n=58) | IDD trait score  below -1.5SD (n=44) |
| Gross motor at 10 months |  |  |  |  |  |
| Below -1SD | 160 | 16 (10%) | 16 (10%) | 21 (13%) | 18 (11%) |
| Within +/- 1SD | 591 | 36 (6%) | 40 (7%) | 31 (5%) | 23 (4%) |
| Above +1SD | 46 | 4 (9%) | 3 (7%) | 3 (6%) | 0 (0%) |
| Visual reception at 10 months |  |  |  |  |  |
| Below -1SD | 180 | 17 (9%) | 18 (10%) | 18 (10%) | 22 (12%) |
| Within +/- 1SD | 548 | 35 (6%) | 36 (6%) | 34 (6%) | 17 (3%) |
| Above +1SD | 68 | 4 (6%) | 5 (7%) | 3 (4%) | 2 (3%) |
| Fine motor at 10 months |  |  |  |  |  |
| Below -1SD | 169 | 19 (11%) | 13 (8%) | 16 (9%) | 16 (9%) |
| Within +/- 1SD | 541 | 33 (6%) | 39 (7%) | 38 (7%) | 24 (4%) |
| Above +1SD | 86 | 4 (5%) | 7 (8%) | 1 (1%) | 1 (1%) |
| Receptive language at 10 months |  |  |  |  |  |
| Below -1SD | 151 | 10 (7%) | 9 (6%) | 12 (8%) | 12 (8%) |
| Within +/- 1SD | 550 | 37 (7%) | 45 (8%) | 35 (6%) | 24 (4%) |
| Above +1SD | 93 | 9 (7%) | 5 (5%) | 7 (8%) | 5 (5%) |
| Expressive language at 10 months |  |  |  |  |  |
| Below -1SD | 217 | 17 (8%) | 12 (6%) | 14 (6%) | 12 (6%) |
| Within +/- 1SD | 487 | 35 (7%) | 45 (9%) | 36 (7%) | 28 (6%) |
| Above +1SD | 84 | 4 (5%) | 2 (2%) | 4 (5%) | 0 (0%) |
| Gross motor at 14 months |  |  |  |  |  |
| Below -1SD | 182 | 18 (10%) | 20 (11%) | 24 (13%) | 18 (10%) |
| Within +/- 1SD | 488 | 34 (7%) | 33 (7%) | 21 (4%) | 19 (4%) |
| Above +1SD | 85 | 5 (6%) | 7 (8%) | 8 (9%) | 2 (2%) |
| Visual reception at 14 months |  |  |  |  |  |
| Below -1SD | 112 | 9 (8%) | 12 (11%) | 13 (12%) | 11 (10%) |
| Within +/- 1SD | 571 | 46 (8%) | 42 (7%) | 35 (6%) | 29 (5%) |
| Above +1SD | 78 | 2 (3%) | 6 (8%) | 4 (5%) | 1 (1%) |
| Fine motor at 14 months |  |  |  |  |  |
| Below -1SD | 131 | 9 (7%) | 17 (13%) | 12 (9%) | 18 (14%) |
| Within +/- 1SD | 561 | 44 (8%) | 41 (7%) | 37 (7%) | 22 (4%) |
| Above +1SD | 68 | 4 (6%) | 3 (4%) | 4 (5%) | 1 (1%) |
| Receptive language at 14 months |  |  |  |  |  |
| Below -1SD | 133 | 6 (5%) | 11 (8%) | 8 (6%) | 9 (7%) |
| Within +/- 1SD | 523 | 44 (8%) | 40 (8%) | 40 (8%) | 27 (5%) |
| Above +1SD | 78 | 5 (6%) | 5 (6%) | 2 (3%) | 1 (1%) |
| Expressive language at 14 months |  |  |  |  |  |
| Below -1SD | 134 | 13 (10%) | 15 (11%) | 13 (10%) | 14 (10%) |
| Within +/- 1SD | 543 | 38 (7%) | 41 (8%) | 35 (6%) | 24 (4%) |
| Above +1SD | 76 | 7 (8%) | 3 (4%) | 3 (4%) | 3 (4%) |
| Gross motor at 18 months |  |  |  |  |  |
| Below -1SD | 116 | 11 (10%) | 13 (11%) | 17 (15%) | 17 (15%) |
| Within +/- 1SD | 578 | 37 (6%) | 44 (8%) | 35 (6%) | 23 (4%) |
| Above +1SD | 107 | 8 (8%) | 3 (3%) | 3 (3%) | 2 (2%) |
| Visual reception at 18 months |  |  |  |  |  |
| Below -1SD | 138 | 12 (9%) | 15 (11%) | 12 (9%) | 19 (14%) |
| Within +/- 1SD | 576 | 41 (7%) | 39 (7%) | 43 (7%) | 23 (4%) |
| Above +1SD | 83 | 4 (4%) | 6 (6%) | 1 (1%) | 1 (1%) |
| Fine motor at 18 months |  |  |  |  |  |
| Below -1SD (n=98) | 98 | 10 (10%) | 12 (12%) | 11 (11%) | 9 (9%) |
| Within +/- 1SD | 616 | 42 (7%) | 43 (7%) | 43 (7%) | 32 (5%) |
| Above +1SD | 91 | 5 (5%) | 5 (5%) | 2 (2%) | 0 (0%) |
| Receptive language at 18 months |  |  |  |  |  |
| Below -1SD | 107 | 12 (11%) | 9 (8%) | 9 (8%) | 11 (10%) |
| Within +/- 1SD | 555 | 39 (7%) | 45 (8%) | 40 (7%) | 29 (5%) |
| Above +1SD | 130 | 5 (4%) | 5 (4%) | 5 (4%) | 1 (1%) |
| Expressive language at 18 months |  |  |  |  |  |
| Below -1SD | 105 | 13 (12%) | 16 (15%) | 18 (17%) | 13 (12%) |
| Within +/- 1SD | 606 | 41 (7%) | 40 (7%) | 35 (6%) | 28 (5%) |
| Above +1SD | 88 | 3 (3%) | 4 (5%) | 3 (7%) | 1 (1%) |
| Gross motor at 24 months |  |  |  |  |  |
| Below -1SD | 160 | 12 (8%) | 24 (15%) | 16 (10%) | 18 (11%) |
| Within +/- 1SD | 518 | 38 (7%) | 30 (6%) | 33 (6%) | 20 (4%) |
| Above +1SD | 113 | 6 (5%) | 5 (4%) | 2 (2%) | 3 (3%) |
| Visual reception at 24 months |  |  |  |  |  |
| Below -1SD | 137 | 15 (11%) | 17 (12%) | 13 (9%) | 18 (13%) |
| Within +/- 1SD | 547 | 39 (7%) | 37 (7%) | 35 (6%) | 22 (4%) |
| Above +1SD | 117 | 5 (4%) | 6 (5%) | 8 (7%) | 2 (2%) |
| Fine motor at 24 months |  |  |  |  |  |
| Below -1SD | 160 | 12 (8%) | 20 (13%) | 20 (13%) | 24 (15%) |
| Within +/- 1SD | 541 | 40 (7%) | 37 (7%) | 33 (6%) | 16 (3%) |
| Above +1SD | 101 | 7 (7%) | 4 (4%) | 3 (3%) | 2 (2%) |
| Receptive language at 24 months |  |  |  |  |  |
| Below -1SD | 119 | 16 (13%) | 17 (14%) | 13 (11%) | 18 (15%) |
| Within +/- 1SD | 583 | 37 (6%) | 39 (7%) | 39 (7%) | 22 (4%) |
| Above +1SD | 76 | 3 (4%) | 3 (4%) | 2 (3%) | 0 (0%) |
| Expressive language at 24 months |  |  |  |  |  |
| Below -1SD | 129 | 12 (9%) | 16 (12%) | 17 (13%) | 16 (12%) |
| Within +/- 1SD | 565 | 45 (8%) | 38 (7%) | 35 (6%) | 22 (4%) |
| Above +1SD | 91 | 2 (2%) | 4 (4%) | 2 (2%) | 2 (2%) |
| Gross motor at 32 months |  |  |  |  |  |
| Below -1SD | 149 | 12 (8%) | 19 (13%) | 20 (13%) | 15 (10%) |
| Within +/- 1SD | 519 | 37 (7%) | 33 (6%) | 32 (6%) | 23 (4%) |
| Above +1SD | 108 | 5 (5%) | 4 (4%) | 1 (1%) | 1 (1%) |
| Visual reception at 32 months |  |  |  |  |  |
| Below -1SD | 137 | 16 (12%) | 19 (14%) | 16 (12%) | 25 (18%) |
| Within +/- 1SD | 555 | 36 (6%) | 36 (6%) | 35 (6%) | 12 (2%) |
| Above +1SD | 100 | 3 (3%) | 3 (3%) | 3 (3%) | 3 (3%) |
| Fine motor at 32 months |  |  |  |  |  |
| Below -1SD | 130 | 16 (12%) | 17 (13%) | 20 (15%) | 21 (16%) |
| Within +/- 1SD | 548 | 39 (7%) | 39 (7%) | 30 (5%) | 18 (3%) |
| Above +1SD | 115 | 1 (1%) | 2 (2%) | 4 (3%) | 1 (1%) |
| Receptive language at 32 months |  |  |  |  |  |
| Below -1SD (n=82) | 82 | 11 (13%) | 13 (16%) | 11 (13%) | 13 (16%) |
| Within +/- 1SD | 604 | 41 (7%) | 38 (6%) | 39 (6%) | 24 (4%) |
| Above +1SD | 103 | 4 (4%) | 6 (6%) | 4 (4%) | 1 (1%) |
| Expressive language at 32 months |  |  |  |  |  |
| Below -1SD | 120 | 15 (13%) | 19 (16%) | 16 (13%) | 19 (16%) |
| Within +/- 1SD | 560 | 38 (7%) | 38 (7%) | 35 (6%) | 19 (3%) |
| Above +1SD | 109 | 3 (3%) | 2 (2%) | 0 (0%) | 2 (2%) |
| Gross motor at 40 months |  |  |  |  |  |
| Below -1SD | 128 | 11 (9%) | 19 (15%) | 21 (16%) | 16 (13%) |
| Within +/- 1SD | 571 | 40 (7%) | 37 (7%) | 31 (5%) | 27 (5%) |
| Above +1SD | 90 | 3 (3%) | 3 (3%) | 3 (3%) | 0 (0%) |
| Visual reception at 40 months |  |  |  |  |  |
| Below -1SD | 141 | 18 (13%) | 18 (13%) | 15 (11%) | 22 (16%) |
| Within +/- 1SD | 529 | 32 (6%) | 34 (6%) | 35 (7%) | 21 (4%) |
| Above +1SD | 133 | 4 (3%) | 6 (5%) | 6 (5%) | 1 (1%) |
| Fine motor at 40 months |  |  |  |  |  |
| Below -1SD | 122 | 18 (15%) | 19 (16%) | 22 (18%) | 26 (21%) |
| Within +/- 1SD | 530 | 37 (7%) | 36 (7%) | 33 (6%) | 16 (3%) |
| Above +1SD | 153 | 2 (1%) | 6 (4%) | 2 (1%) | 2 (1%) |
| Receptive language at 40 months |  |  |  |  |  |
| Below -1SD | 96 | 14 (15%) | 16 (17%) | 10 (10%) | 17 (18%) |
| Within +/- 1SD | 588 | 37 (6%) | 37 (6%) | 39 (6%) | 25 (4%) |
| Above +1SD | 121 | 5 (4%) | 6 (5%) | 7 (6%) | 1 (1%) |
| Expressive language at 40 months |  |  |  |  |  |
| Below -1SD | 121 | 10 (8%) | 17 (14%) | 17 (14%) | 23 (19%) |
| Within +/- 1SD | 554 | 44 (8%) | 38 (7%) | 34 (6%) | 21 (4%) |
| Above +1SD | 123 | 3 (2%) | 5 (4%) | 6 (5%) | 0 (0%) |

ADHD trait: inverted Z-converted ADHD-RS percentile score. ASD trait: inverted Z-converted SRS-2 total T-score. DCD trait: Z-converted DCDQ total score. IDD trait: inverted Z-converted full-scale IQ, all measured at age 9 years.

Figure S1. The distributions of outcome measures at age 9: a) ADHD trait: inverted Z-converted ADHD-RS percentile score, b) ASD trait: inverted Z-converted SRS-2 total T-score, c) DCD trait: Z-converted DCDQ total score, d) IDD trait: Z-converted full-scale IQ.

d)

c)

b)

a)


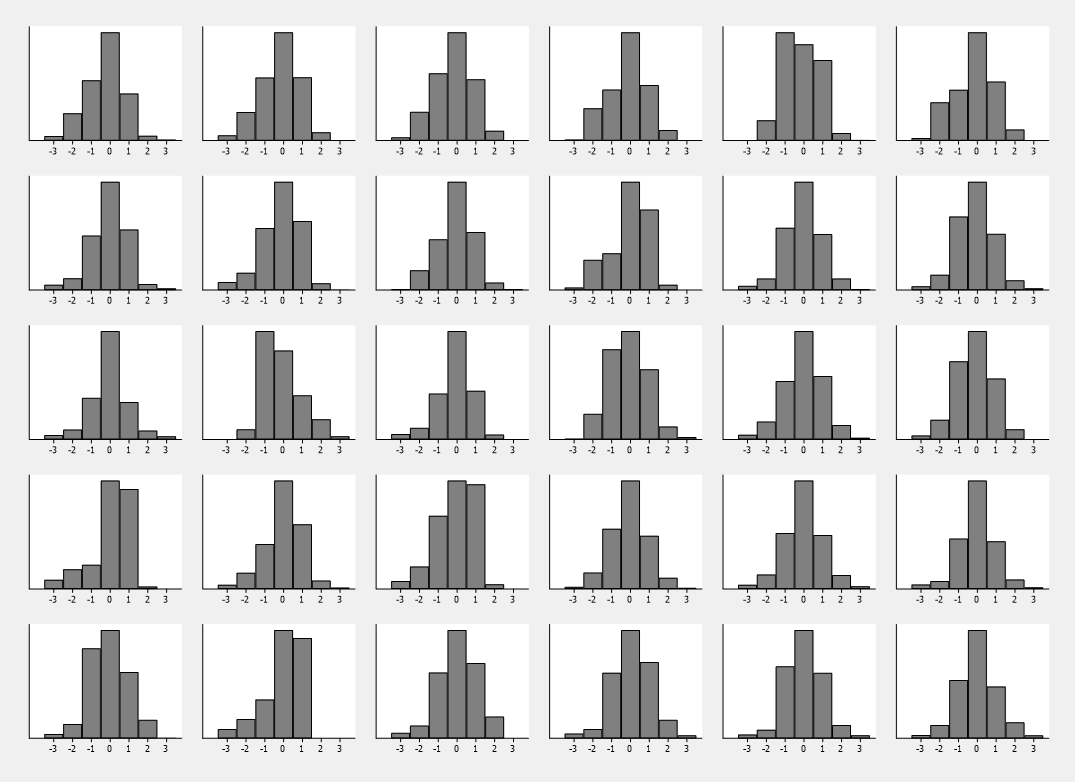
Figure S2. The distributions of neurodevelopmental measures: Z-scores of gross motor, visual reception, fine motor, receptive language, expressive language domains at ages 10, 14, 18, 24, 32, and 40 months using the Mullen Scales of Early Learning.

Gross motor motor

Visual

reception

Fine motor motor

Receptive

language

Expressive

language

10 months

14 months

18 months

24 months

32 months

40 months
